# Correlation between Allergic Rhinitis and Otitis Media with Effusion

**Published:** 2019-07

**Authors:** Mohammad Reza Sharifian, Mahmoud Mahmoudi, Babak Pourmomenarabi, Mohammad Reza Keramati

**Affiliations:** 1 *Department of Otorhinolaryngology-Head & Neck Surgery, Mashhad University of Medical Sciences, Mashhad, Iran.*; 2 *Immunology Research Centre, Imam Reza Hospital, Faculty of Medicine, Mashhad University of Medical Sciences, Mashhad, Iran.*; 3 *Cancer Molecular Pathology Research Center, Imam Reza Hospital, Faculty of Medicine, Mashhad University of Medical Sciences, Mashhad, Iran.*

**Keywords:** Allergic rhinitis, Nasal mucosa, Otitis media with effusion

## Abstract

**Introduction:**

Otitis media with effusion (OME) is prevalent among children in such a way that it is the most common cause of hearing loss and surgery in childhood. Immunoglobulin E (IgE) mediated hypersensitivity has been proposed as a causative factor in the development of OME; however, there has been contrasting data in this regard. Therefore, the present study aimed to detect the possibilities of interconnection.

**Materials and Methods:**

In this study, 37 OME children were selected as the case group and 52 children were randomly chosen as the control group. Allergic rhinitis prevalence, serum total IgE concentration, serum eosinophil count, and nasal scraping cytology were evaluated in all the children. Furthermore, the skin prick test was performed in the OME group and suspected allergic rhinitis patients in the control group.

**Results:**

Allergic rhinitis prevalence was notably higher among OME patients than in the control group (P*=*0.01). There were no remarkable differences in eosinophil counts and serum IgE concentrations in the two groups. Nasal smear eosinophils did not show any significant difference between the two groups; however, Appreciable difference was observed in the allergic rhinitis patients, compared to other OME patients (P*=*0.004).

**Conclusion:**

There may be a correlation between allergic rhinitis and development of OME. Therefore, it seems reasonable to examine allergic rhinitis patients for OME.

## Introduction

Otitis media with effusion (OME) is the collection of fluid within the middle ear space behind a nonperforated eardrum due to inflammatory processes but without any features of acute inflammation (1). OME is prevalent among children with a rough incidence of 20%, and it is the most common cause of hearing loss and surgery in childhood (2-4). Although in most cases OME is self-limited and improves spontaneously and resolves as the child grows, in about 5% of patients even surgery will not prevent the injury to the middle ear (5).

Heterogeneous factors can participate in the origins of OME. Some of them are congenitally acquired, including Down syndrome, as well as cleft palate and cystic fibrosis, which are associated with the increased probability of developing OME (6). Viral and bacterial infections play an important role in the pathophysiology of serous otitis media.Cultures of middle ear secretions demonstrated bacterial proliferation in 28-43% of the subjects, and 16-19% of the cases displayed viral infection (7).

It has been a matter of debate whether immunoglobulin E (IgE) mediated reactions could be implicated in the development of OME or not. The results of clinical studies have shown contrasting data in this regard. Several studies have estimated 80% of allergy incidence in OME patients while some others have detected nearly no correspondence at all (8-14). Elevated concentration levels of total and specific IgE have been detected in some studies whereas other clinical practices have noticed different results (13-17).

According to the literature, the data related to atopic children have demonstrated a preponderance of bilateral OME and a more profound hearing loss, compared to nonatopic subjects (18, 19). In fact, getting cvahildren prescribed with an antibiotic in combination with anti-allergy agents have been shown to be more influential than exclusively the use of antibiotics in allergic OME patients (20). Therefore, it seems reasonable to ascertain whether a patient with persistent or recurrent OME suffers from allergic conditions or not to develop a treatment plan. Consequently, the present study was carried out to assess the correlation between OME and allergy.

## Materials and Methods

A total of 37 OME children (28 boys and 9 girls), who underwent ventilation tube insertion due to the longevity of effusion for more than 3 months or because of recurrent OME or language developmental disorders, were enrolled in the patient group between November 2015 and December 2016. The patients in the case group were from 3 to 15 years of age, and OME was diagnosed by otoscopic examination or a B tympanogram on impedance audiometry. 

In addition, 52 children were selected (30 boys and 22 girls) within the age range of 2-14 years (6.13±3.24 years) without any history or clinical findings of OME. All the subjects in the control group were chosen from those who were visited in our clinic in an outpatient setting for reasons other than epistaxis or complaints related to adenotonsillar hypertrophy or tonsillitis during the same period. An ear, nose, and throat (ENT) specialist examined all the children with respect to the permission of their parents. Children with predisposing conditions, including head and neck anomalies and cleft palate or Down syndrome were not included in the study. 

Modified Asthma and Allergy in childhood (ISAAC) questionnaire was filled out by the parents of all the children (21). Allergic rhinitis was established through a positive answer to the following core question: Have your child ever had a problem with sneezing, a runny or blocked nose not associated with a cold or the flu? 

Pale and/or swollen mucosa and turbinates on examination could confirm the diagnosis. None of the children received antihistaminic treatment one week before and at the time of the study. Anterior rhinoscopy was performed, and all the children underwent nasal scraping for cytology evaluation.Blood samples of the patients and control subjects were analyzed for serum IgE concentration and serum eosinophil counts. 

All the children in the control group were selected from individuals with sufficient blood taken for IgE level determination after reading complete blood count for another reason. The parents were fully explained about the purpose of the study and written informed consent was obtained from the parents. 

**Nasal cytology**

During anterior rhinoscopy, Cells were collected by a light brushing of the middle third of the inferior turbinate with a cotton bud. The specimen was then air-dried on a glass slide and stained with Giemsa's solution. The samples then were evaluated for the presence of inflammatory cells, including eosinophils, neutrophils, basophils, as well as bacteria and spores. At least 10 fields were observed from each slide under magnification of 400x. The count of each cell type was then conveyed as a percentage of the total cells (including mucinous and ciliated cells), and a score was assigned to each cell type according to (Table.1) (22).

**Table 1:** Quantitative and descriptive grading for nasal cytology reporting

	**Grading**	**Quantitative**	**Description**
Neutrophils and eosinophil	None	0	0
	Occasional	0.1-1%	½+
	Few scattered cells, small clumps	1.1-5%	1+
	Moderate number, large clumps	5-15%	2+
	Large clumps not covering the field	15-20%	3+
	Clumps covering entire field	>20%	4+
Bacteria and spores	None observed	None standardized	0
	Occasional clumps		1+
	Moderate number		2+
	Many cells easily seen		3+
	Bacteria/spores over the entire field		4+

**Skin prick test**

The skin prick test was performed on the forearm of all the children in the patient group and suspicious allergic rhinitis subjects with a positive answer to the modified ISAAC questionnaire in the control group using allergenic extracts as listed in Table 2. All the extracts were purchased from GREER (GREER,NC,USA). Solutions of glycerinated histamine phosphate (5 mg/ml) and glycerosaline were used as positive and negative controls, respectively. Wheals with at least 3 mm wide were considered as positive. Provided that there was a history of taking antihistamine in the previous 72 h, the prick test was carried out again later on. 

**Table 2:** allergy extracts used for prick testing

	**Amaranthus retroflexus**	**Chenopodium album**	**Salsola kali**
Weeds	Ambrosia trifida	Kochia scoparia	Urtica dioica
	Artemisia vulgaris	Plantago lanceolata	Xanthium strumarium
	Atriplex polycarpa	Rumex acetosella	
Mites	Dermatophagoides farinae	Dermatophagoide pteronyssinus	
		

**Statistics**

All the statistical analyses were performed using SPSS software (version 11.5). The Kolmogorov-Smirnov test was used to test variants of normality. The student’s t-test was used to collate normal distribution variants. The Mann-Whitney U test was utilized for other variants. The Chi-squared test was used to compare the numeric variants. We took advantage of the univariate analysis to adjust the age variable, and no difference was observed with the final results. *P-*value less than 0.05 was considered statistically significant.

## Results

The two groups were matched with regard to gender distribution based on the Chi-squared test (P=0.08). Despite the fact that the subjects in the control group were older than OME patients, the results of the student’s t-test did not show a considerable difference between the two groups (t=2.47, P=0.015). Out of the 37 patients with OME, 9 (24.3%) patients had allergic rhinitis while 3 out of 52 subjects in the control group ,(5.8%) cases, had allergic rhinitis, which showed a significant difference in the incidence of allergic rhinitis (Table 3; P=0.01).

**Table 3:** Demographic characteristics

	**OME children group**	**Control group**	**P value**
Numbers	37	52	-
Age± SD (range)	7.78± 2.9 (3-15years)	6.13± 3.24 (2-14years)	-
Male: Female	28:9	30:22	-
(Accompanying condition)			
Allergic Rhinitis	9(24.3%)	3(5.8%)	0.01
Unilateral:Bilateral effusion	7:30	-	-

No significant difference was observed between the patient and control groups regarding the elevated serum IgE levels (>100IU/ml; 11 [29.7%] vs 7 [13.5%]; P=0.06). The mean values of serum IgE concentration were 124.7±180.44 and 42.57±80.53 IU/ml in the patient and control group, respectively (P-value was not significant). The average eosinophil count was 270.76±231.22 and 317.02 ±207.45 mm^3^ in the patient and control groups, respectively, and the difference was not significant (P*=*0.1). Seventeen (45.9%) children with OME had at least one positive skin prick test to any antigen.

The results of medical examination and skin prick test affirmed the presence of allergic rhinitis in 9 out of 13 positive questionnaires in the OME group and all the 3 subjects in the control group. The male/female ratio was equal to 3.1 that indicated a statistically significant difference (P*=*0.002). Unilateral effusion was observed in 7 (41.6%) patients, and 2 of the cases were diagnosed with allergic rhinitis.

A positive finding for nasal smear eosinophils did not show a significant difference between the OME group and control group (Table.4;P*=*0.056). Moreover, no significant difference was observed for nasal eosinophil scores (Table.5; P*=*0.18). In contrast, a significant difference was observed for neutrophilic granulocytes and bacteria. Data for metachromatic cells were not included in the analysis because these cells were only occasionally observed. The presence of eosinophils in the nasal smear was significantly associated with allergic rhinitis (P=0.004).

**Table 4:** Nasal cytology in children with OME and in the control group

**Cell type**	**OME group** **(n=32)**	**Control group** **(n=51)**	**P-value**
Eosinophil	6	5	0.056
Neutrophil	22	43	0.007
Bacteria	4	15	0.007

**Table 5:** Mean nasal cytology score

**Cell type**	**OME group** **(n=32)**	**Control group** **(n=51)**	**P-value**
Eosinophil	0.69	0.14	Not significant
Neutrophil	2.31	3.07	0.03
Bacteria	0.22	0.62	Not significant

## Discussion

The incidence of allergic rhinitis in the children population has been reported 5-10% while surveys have estimated the incidence of allergic rhinitis from 14-89% among children with OME (23,24). The results of the present study demonstrated that 24.3% (9 out of 37) of the patients had allergic rhinitis whereas 5.8% of the subjects in the control group were diagnosed with allergic rhinitis, which represented a significant difference between the two groups of the study (P*=*0.01). To avoid the risk of bias as a result of a lack of well-defined diagnostic criteria for allergic rhinitis, the modified ISAAC questionnaire was introduced into the study to standardize the case definition and maximize the study value. Moreover, the questionnaires were matched with a medical examination to validate them more.

Several mechanisms are proposed for the role of allergy in the development of otitis media, including inflammatory swelling and obstruction of eustachian tube orifice, the middle ear as a shock organ, and obstruction of the nose due to inflammation, and aspiration of bacterial contaminated allergic nasopharyngeal secretions into the middle ear (25).

In the present study, bacteria positive nasal smears showed a significant difference between the OME and control groups yet to the benefit of the control group (P*=*0.007), which was one major drawback of this study. Due to the ethical issues, control subjects were recruited from outpatient referrals for laboratory testing, and the comparison with a completely healthy control group seems sensible. Moreover, the similar observed significant difference between the two groups in terms of neutrophilic positive nasal smears (P*=*0.007) and neutrophilic scores (P*=*0.03) may imply bacterial inflammation of the upper respiratory tract in the control group subjects. 

Despite the fact that the results of this study did not show considerable differences between the two groups regarding the findings of eosinophils at the site of the nasal mucosa and contrary to the results of a study conducted by Caffarelli et al, (26), the comparison of nasal eosinophil scores showed 4+ score in 5 out of 6 the OME patients (83%) while just one control subject had 4+ score (20%). In addition, none of the others showed nasal eosinophil score of more than 1+ that was indicative of higher nasal eosinophil scores in the OME group. A significant difference was observed in the variants of nasal eosinophilia and scores in the patients diagnosed with allergic rhinitis, compared to other OME patients (P*=*0.004). 

The above-mentioned findings indicated that allergic inflammation may play a pathogenic role in a group of OME patients. We used skin prick testing to confirm the diagnosis of AR in OME patients and in control group subjects with a positive response to modified ISAAC questionnaire, yet the observed 28.5% positivity of prick testing in the none allergic rhinitis OME patients, put emphasis to the fact that a positive skin testing result alone, does not verify the diagnosis of AR in the absence of a supporting clinical history.

Total IgE is elevated in 30-40% of the patients with allergic rhinitis and can be raised in patients with nonallergic conditions and in normal subjects which makes this parameter not useful in the diagnosis of allergic rhinitis (27). High serum IgE levels (100IU/ml) were evaluated in 29.7% and 13.5% of the OME and control groups, respectively, which did not show a significant difference. However, 81.8% of the OME patients with elevated serum IgE levels showed positive results of a skin prick test. Furthermore, no significant difference was observed in total serum IgE concentration between the two groups. Nonetheless, serum IgE concentrations were higher in allergic rhinitis patients among other OME patients.

Although eosinophils basically function against parasitic infections, if activated inappropriately, they may be harmful to body tissues and can induce inflammation. In addition, no statistically significant difference was noticed between OME and control groups regarding eosinophil counts. The normal blood counts of eosinophils are in the range of 0-240 mm^3^ with 95% confidence limits to fall between 15 and 650 mm^3^ eosinophils (28, 29). Other causes of eosinophilia, such as parasitic diseases, drug hypersensitivity, and eosino-philia-myalgia may be an explanation for some high eosinophil counts in the control group.

## Conclusion

Allergic rhinitis was significantly higher among pediatric OME patients than those without OME. On the other hand, these two groups showed no significant differences in eosinophil counts and serum IgE concentration. Though nasal smear eosinophils did not demonstrate a significant difference between the OME and control groups, a significant difference was observed between the allergic rhinitis patients, compared to other OME patients.
